# The Collaborative Cross-Mouse Population for Studying Genetic Determinants Underlying Alveolar Bone Loss Due to Polymicrobial Synergy and Dysbiosis

**DOI:** 10.3390/ijms25010473

**Published:** 2023-12-29

**Authors:** Aysar Nashef, Rawan Qabaja, Ronen Hazan, Arne Schafer, Hatice Hasturk, Alpdogan Kantarci, Yael Houri-Haddad, Fuad A. Iraqi

**Affiliations:** 1 Department of Prosthodontics, Faculty of Dental Medicine, Hebrew University, Jerusalem 9112102, Israel; dr.aysarn@gmail.com (A.N.); domo-3-2009@hotmail.com (R.Q.); yaelho@ekmd.huji.ac.il (Y.H.-H.); 2 Department of Oral and Maxillofacial Surgery, Meir Medical Center, Kfar Saba 4428164, Israel; 3 School of Medicine, Tel Aviv University, Tel Aviv 6997801, Israel; 4 Institute of Biomedical and Oral Research (IBOR), Faculty of Dental Medicine, Hebrew University of Jerusalem, Jerusalem 9112102, Israel; ronenh@ekmd.huji.ac.il; 5 Department of Periodontology and Synoptic Dentistry, Institute for Dental and Craniofacial Sciences, Charité–Medical University, 10117 Berlin, Germany; arne.schaefer@charite.de; 6 The Forsyth Institute, Applied Oral Sciences, Cambridge, MA 02142, USA; hhasturk@forsyth.org (H.H.); akantarci@forsyth.org (A.K.); 7 Department of Clinical Microbiology and Immunology, Sackler Faculty of Medicine, Tel Aviv University, Tel Aviv 6997801, Israel

**Keywords:** periodontitis, microbiome, dysbiosis, collaborative cross (CC) mouse, 16srRNA sequencing, computerized microtomography (micro-CT)

## Abstract

Dysbiosis of oral microbiota is associated with the initiation and progression of periodontitis. The cause-and-effect relationship between genetics, periodontitis, and oral microbiome dysbiosis is poorly understood. Here, we demonstrate the power of the collaborative cross (CC) mice model to assess the effect of the genetic background on microbiome diversity shifts during periodontal infection and host suitability status. We examined the bacterial composition in plaque samples from seven different CC lines using 16s rRNA sequencing before and during periodontal infection. The susceptibility/resistance of the CC lines to alveolar bone loss was determined using the micro-CT technique. A total of 53 samples (7 lines) were collected before and after oral infection using oral swaps followed by DNA extraction and 16 s rRNA sequencing analysis. CC lines showed a significant variation in response to the co-infection (*p* < 0.05). Microbiome compositions were significantly different before and after infection and between resistant and susceptible lines to periodontitis (*p* < 0.05). Gram-positive taxa were significantly higher at the resistant lines compared to susceptible lines (*p* < 0.05). Gram-positive bacteria were reduced after infection, and gram-negative bacteria, specifically anaerobic groups, increased after infection. Our results demonstrate the utility of the CC mice in exploring the interrelationship between genetic background, microbiome composition, and periodontitis.

## 1. Introduction

Periodontitis is one of the most common chronic infectious diseases worldwide [1]. Untreated severe periodontitis cases may cause tooth loss. It is evident that susceptibility to periodontitis is attributed to an interplay of the host genetic background with environmental factors and disturbances of the oral microbiome [1,2,3,4,5,6]. Accordingly, periodontitis is regarded as a “dysbiositic disease”. Dysbiosis is a condition in which the balanced state of the ecosystem is disturbed. The hypothesis suggests that the transition from periodontal health to disease reflects a significant alteration in the number and community organization of the oral commensal bacteria in the periodontal pocket. This shift in the microbial community composition leads to alterations in the host–microbe crosstalk sufficient to mediate destructive inflammation and bone loss [7,8]. Different studies suggested that this shift may be mediated by environmental factors and/or keystone pathogens such as *Porphyromonas gingivalis* (*P.g.*) [9,10,11,12,13,14].

In the last decades, numerous studies evaluated the alterations in oral microbiota composition across different stages of periodontal disease. However, only a few studies have assessed the cause-and-effect relationship between periodontal disease severity, the genetic constitution of the host, and alterations in the composition of the oral microbiota. Using appropriate animal models could overcome these limitations and contribute significantly to an improved understanding of the relationship between alterations in the composition of the oral microbiota and the genetic architecture of periodontitis susceptibility.

The ideal mouse model to study complex diseases with complex etiologies mimics the complex genetic diversity among populations and enables the fine mapping of quantitative trait loci (QTLs) underlying defined complex phenotypes [15]. In recombinant inbred line (RIL) crosses of genetically defined strains, chromosomal regions responsible for the genetic variance of complex traits can be mapped as QTLs under defined conditions [16,17,18,19]. To address these requirements, a new genetic resource population, named the collaborative cross (CC) mouse model, was proposed by community efforts.

The CC is a RIL mouse population that was specifically designed for high-resolution QTL mapping. It was created from a full reciprocal mating of five classical inbred strains (A/J, C57BL/6J, 129S1/SvImJ, NOD/ShiLtJ, and NZO/HlLtJ) and three wild-derived strains (CAST/EiJ, PWK/PhJ, and WSB/EiJ) to capture a much greater level of genetic diversity than existing mouse genetic reference populations (GRPs) (Collaborative Cross Consortium 2012). Recently, we showed that CC lines respond differently to experimental periodontitis 42 d after mixed infection with *P.g.* and *Fusobacterium nucleatum* (*F.n.*) and enabled the mapping of confined QTLs that confer susceptibility to alveolar bone loss [3,20].

In the current study, we used the oral mixed-infection model of two periodontitis-related anaerobic bacteria, *P.g.* and *F.n.*, to induce dysbiosis of the oral microbiota. We subsequently explored differences in microbiome composition and periodontal disease development in different CC lines with different genetic backgrounds.

## 2. Results

### 2.1. Susceptibility of CC Lines to Alveolar Bone Volume Affected by Oral-Mixed Infection 

**Figure 1:**
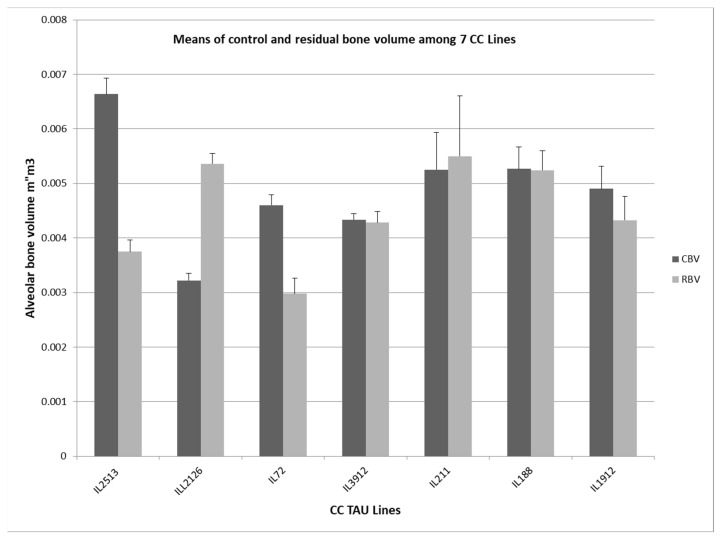
Alveolar bone volume parameters of seven different CC RILs. The figure shows a comparison between means of the alveolar bone volume in each line (±SEM) evaluated using µCT. Gray columns are related to the mean of the control bone volume (CBV), whereas black columns are related to the residual bone volume (RBV).

CC lines showed a significant variation in their response to the co-infection. Based on the one-way ANOVA, two lines (IL72, IL2513) showed a significant reduction in the mean of bone volume level after infection compared to the control group and were considered susceptible lines (*p* < 0.05), while four lines (IL211, IL188, IL1912, and IL3912) did not show significant bone loss after infection and were considered to be resistant lines (Figure 1). Noteworthy, line IL2126 showed a significant bone gain following the infection and is considered an over-resistant line.

### 2.2. Challenge Effect on Microbiome Compositional Shift 

**Figure 2:**
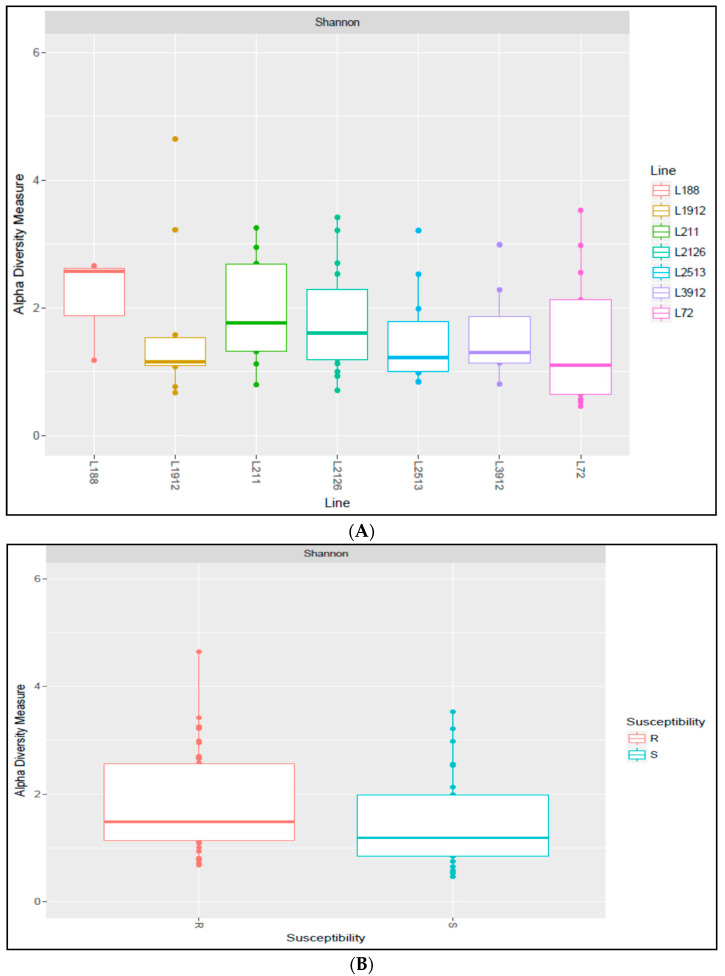

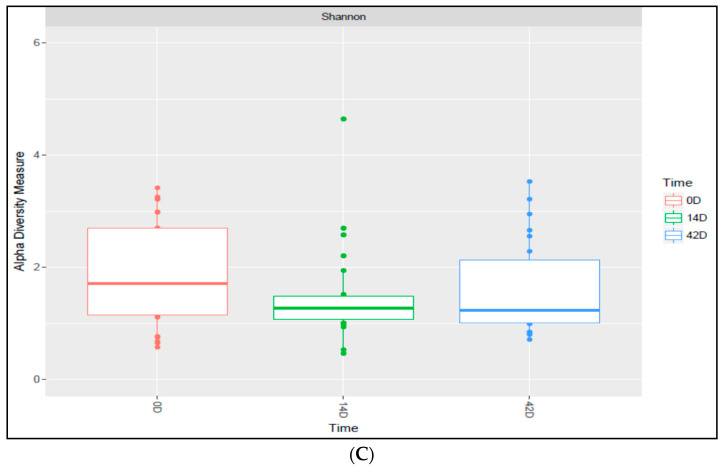
Alpha diversity measure by line, susceptibility status, and time (**C**). (**A**) Alpha diversity comparison based on Shannon’s diversity index. X-axis represents the alpha measurements, and Y-axis represents the TAU RILs. Each box color refers to a line. The results were not significant between lines. (**B**) X-axis represents the alpha measurements, and Y-axis represents susceptibility. Resistant (red) and susceptible (blue), the results were not significant. (**C**) X-axis represents the alpha measurements, and Y-axis represents time. 0 day (red), 14 day (green), and 42 day (blue), the results were not significant.

**Figure 3:**
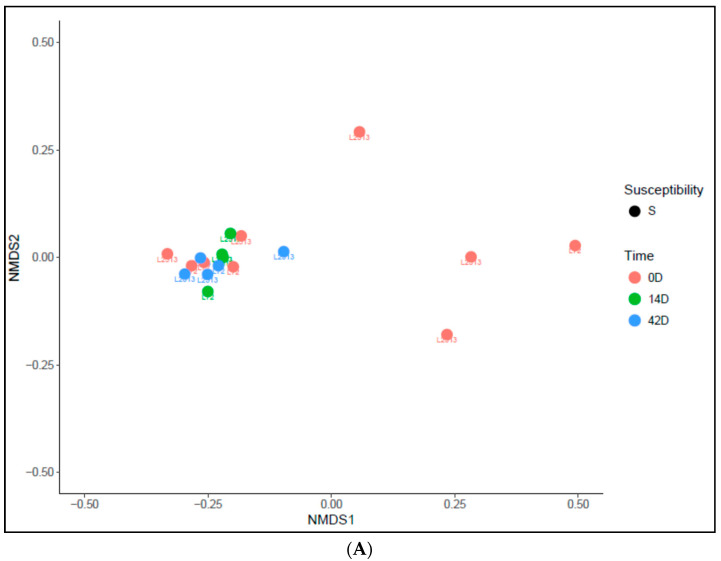

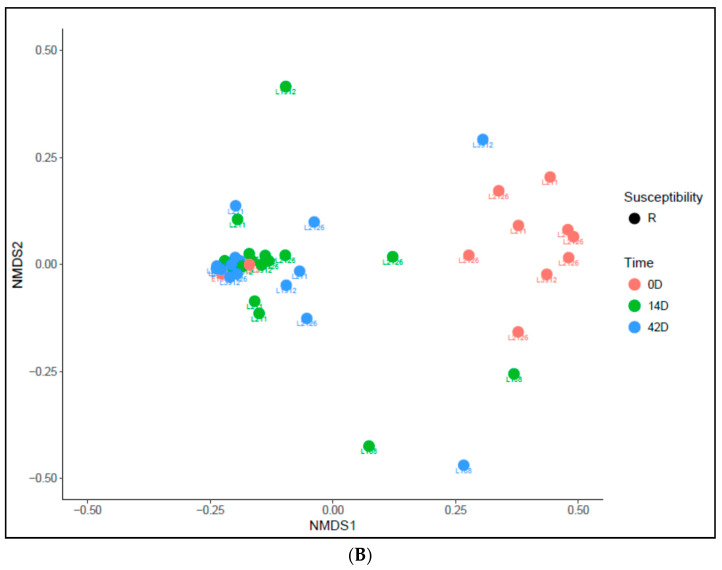
Beta-diversity visualized using a non-metric multidimensional scaling (NMDS) plot. NMDS plots on rank order distances were used to assess the significance of bacterial community composition between different time points. Significant changes (*p* < 0.001) in the species diversity were observed among the seven CC lines due to infection (between time point 1 and the other time points), regardless of their susceptibility status (i.e., susceptible (**A**) and resistant (**B**)). However, no significant change was observed between day 14 and day 42.

Alpha diversity was calculated for different variables at different levels, including susceptible and resistant lines, and at different time points (day 0, day 14, and day 42), including before and after infection. All of these comparisons were not significant (Figure 2). Beta diversity was calculated to assess the change in the diversity of species after infection for each line. A significant change in the species diversity was observed among the seven CC lines after infection (between time point one and the others time points), regardless of their susceptibility status (i.e., susceptible and resistant). However, no significant change was observed between day 14 and day 42 (Figure 2B and Figure 3A).

### 2.3. Microbiome Composition in Control Mice 

**Figure 4:**
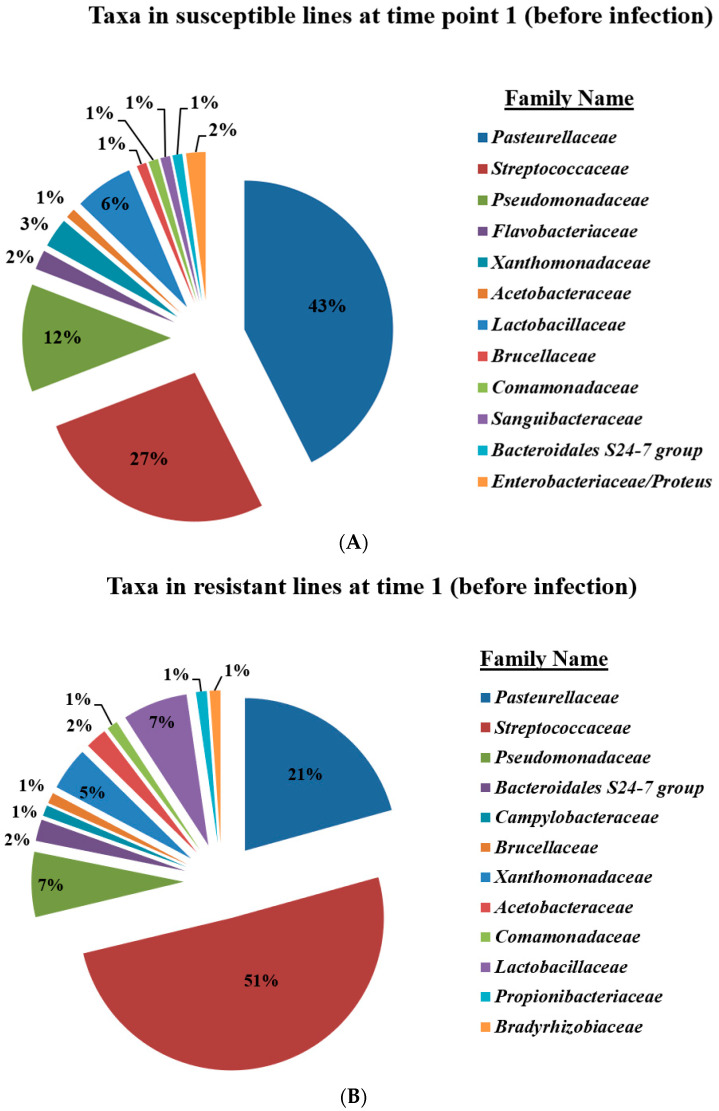
Microbiome composition of susceptible (**A**) and resistant (**B**) RILs at time point 1.

Bacterial composition before infection (point time 1): *Pasteurella* and *Streptococcus* were the majority of the microbiome taxa for both susceptible and resistant lines. In addition, gram-positive taxa were significantly higher at the resistant lines compared to susceptible lines. In addition, the *Streptococcus genus* represented ~50% of the microbiome composition compared to ~25%. Some of the taxa were exclusive for susceptible lines (i.e., *Saguibacteraceae*) and were not shown in the flora of resistant lines, while others) i.e., *Bradyrhizohiaceae* and *Bradyrhizobium*) were present only in the resistant lines (Figure 3B and Figure 4A).

**Figure 5:**
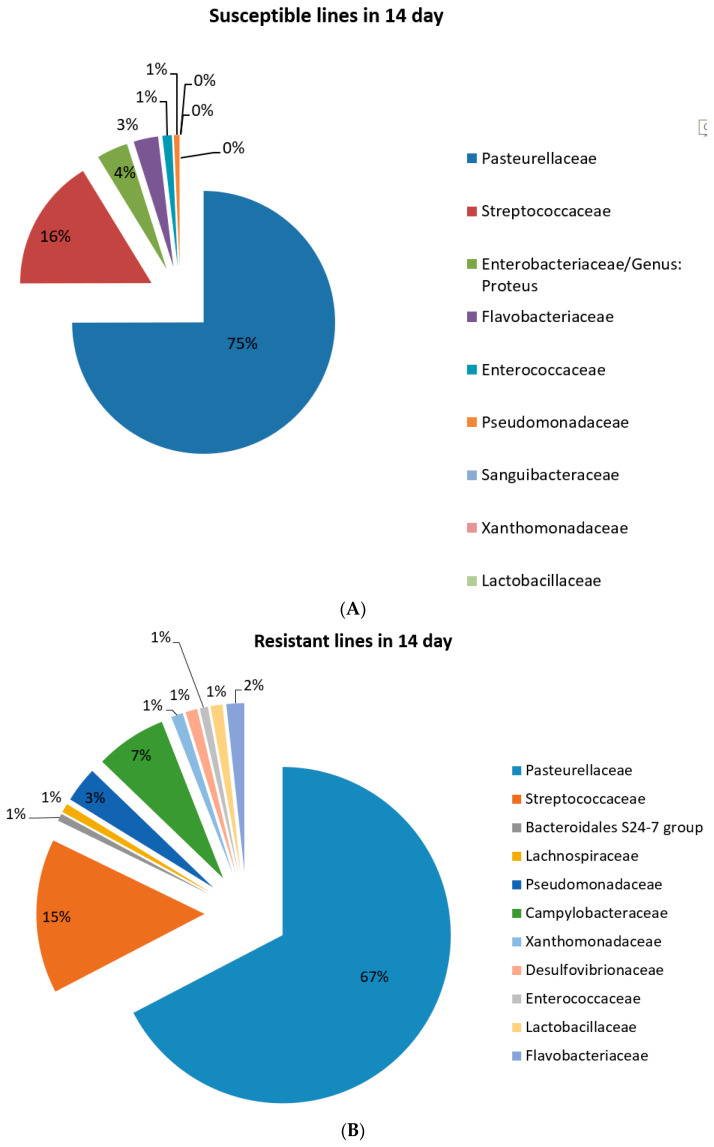
The figure shows the microbiome composition in susceptible (**A**) and resistant (**B**) RILs 14 days after oral mixed infection.

A total of 14 days after the mixed infection (point time 2), the microbiome composition for both susceptible and resistant mice was similar for the two major bacterial groups (*Pasteurella* and *Streptococcus)* but with significant proportional changes compared to time point 1. In addition, the bacteria are mainly gram-negative for both susceptible and resistant lines. Interestingly, the mixed cultures of *P.g.* and *F.n.* were not shown at this stage of post-infection status (Figure 4B and Figure 5A).

**Figure 6:**
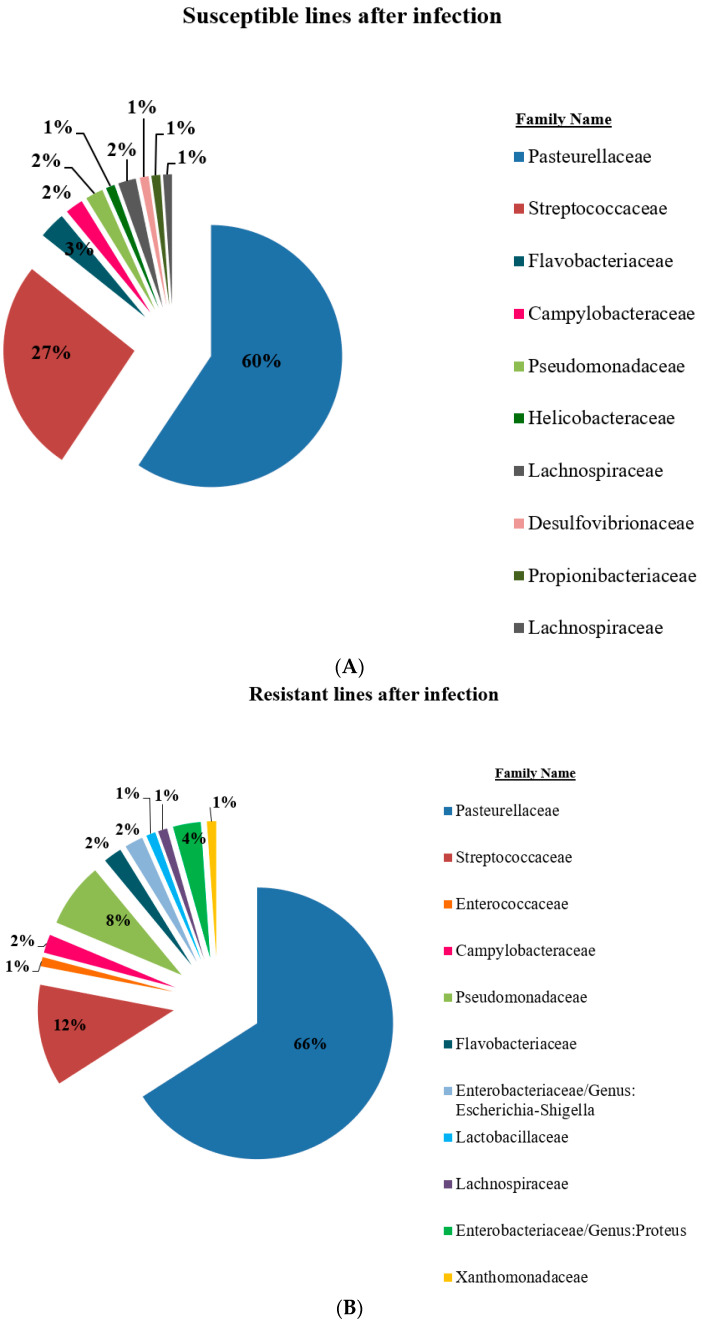
The figure shows the microbiome composition in susceptible (**A**) and resistant (**B**) RILs 42 days after oral mixed infection.

After 42 days of infection (point time 3), there was no significant change compared to 14 days after infection (time 2 point). Microbiome compositional changes were shown between resistant and susceptible lines; while some bacteria showed mainly in the susceptible lines, others were mainly present in the resistant ones. However, these changes were not significant (Figure 5B and Figure 6A).

### 2.4. Correlation between Alveolar Bone Loss and Dysbiosis 

The abundance of *Pasteuerlla* and *Bergeyella* was positively correlated with bone loss in the susceptible lines (r = 0.78, 0.75, *p* < 0.0.5), suggesting a role in bone loss severity after infection, while *Streptococcus* and *Pseudomonas* show a negative correlation with bone loss (r= −0.79, −0.84, *p* < 0.0.5); the lower the number of bacteria is, the worse the disease severity was observed. This suggests the role of these bacteria in keeping bone volume levels healthy.

## 3. Discussion 

The micro-CT results showed variations in the response of different CC lines to infection. Because the RILs were kept within the same controlled environment, we consider that this is related to the genetic differences between the RILs, which we assume are responsible for individual differences of the RILs in susceptibility to periodontal disease. Likewise, a previous study of our lab showed that the heritability tests for 23 CC lines that were challenged using the oral mixed infection model were 0.2. This implies that the variation in host susceptibility to the disease is influenced by genetic factors, as shown by the different phenotypes among the different RILs. For humans, twin studies estimated a heritability of 50% for periodontitis [21]. Each CC line responded differently to the infection according to the type and number of the SNPs in multiple candidate genes. Therefore, the phenotypes showed variation from mild to severe periodontitis or bone formation, which appeared in two CC lines instead of bone decreases. As we know, bone loss is a feature of the disease, but this new phenotype shows how genetic variability affects different genetic pathways, resulting in increased bone formation or resorption in response to bacterial challenge [3,22]. These results validate the power of the CC lines population for studying host genetic susceptibility to complex diseases with complex etiologies.

The 16 s microbiome sequencing showed that the beta diversity was distinctly different before and 42 days after infection. We also observed that resistant lines had a different microbiome compared to susceptible lines before infection. We consider this a valuable result because it gives some insight into how genetic predisposition shapes the microbiome and possibly indicates microbial compositions associated with resistance to infection-induced bone loss. In resistant lines, the gram-positive bacteria formed more than half of the microbiome compositions. This flora is associated with periodontal healthy tissues. It can influence the prevention of pathogenic colonization in different ways, e.g., by limiting the ability of pathogenic bacteria to adhere to appropriate tissue surfaces or by producing metabolic factors that are adverse to periodontal pathogens. After infection, gram-negative bacteria become dominant relative to the gram-positive bacteria, which may increase the susceptibility to periodontitis, resulting from specific virulence factors, which may consist of proteolytic enzymes that break down host tissue and may result in gingival inflammation, loss of gingival attachment, periodontal pocket formation, and alveolar bone and teeth destruction [23,24]. These results may support previous reports that showed that the composition and function of the indigenous oral microbiome may determine an alteration of the symbiotic interaction between the oral microbial community and the host with consequences for the oral and general health of the individual. The alteration of this finely tuned equilibrium between host and hosted microbes allows pathogenic bacteria to manifest their disease-promoting potential and determinate pathological conditions. Moreover, our results may fall in agreement with previous studies, which showed that the dynamic interactions between the various microbial and host factors that drive periodontal tissue destruction are not related to a limited number of periodontopathogenic species but are the outcome of a synergic action of dysbiotic microbial communities in the specific individual [10]. For example, *P.g.*, one of the major etiologic microbial agents of periodontitis that we used in the current study and included in the Socransky Red Complex, requires iron and protoporphyrin IX from heme to survive and support dysbiosis initiation and development and the consequent onset of periodontal disease [25]. However, the changes in microbial diversity between health (eubiosis) and periodontal disease (dysbiosis) remain controversial since some researchers reported a loss of microbial diversity, others indicated an increasing level of microbial diversity, and still others did not report significant differences [26,27,28].

Interestingly, gram-positive bacteria were high in resistant lines before infection, and the *Streptococcus genus* represented half of the microbiome composition. Noteworthy, this genus is well known for its critical role in preventing pathogenic periodontal bacteria colonization (colonization resistance) and was proposed previously as a guided pocket recolonization approach as an alternative to the armamentarium of treatment options for periodontitis [29,30]. After infection, gram-positive bacteria decreased while gram-negative bacteria increased. These bacteria were not associated with alveolar bone loss, which may be related to many reasons. The bacteria did not produce a destructive host response, or microorganisms may lack some virulence factors responsible for periodontal tissue destruction. Notably, we observed that after infection, the genus *Pseudomonas* was reduced in susceptible but not resistant lines. Although speculative, this may indicate a protective role for periodontal tissue destruction.

Noteworthy, while this study demonstrates how the specific genetic background of the RIL shapes characteristics of the immune system and microbiome composition and may be used as a valuable tool for the dissection of the complex relationship, some limitations should be pinpointed. First, the microbiome composition in the current mouse population may not resemble the “natural” relationship of periodontitis to oral microbiome. This is because the dental plaque microbial community that forms on the supragingival area, which was collected in the present study, differs from the subgingival community, which is supposed to be more relevant to periodontal disease. However, collecting microbial samples from the periodontal pocket in the mouse model is almost unachievable. Second, during the sampling process, the samples were not always taken from the same mouse during the 3 points but from the same status and line and were not taken immediately after antibiotic treatment, which may have led to the loss of important information. Finally, in the current study, a small number of mice/lines were used to assess the utility of this model for exploring such complex phenotype; however, to dissect such a complex cause-effect relationship, we need to assess many more lines and mice per line.

In summary, microbiome analysis provides information regarding the differences in microbiome composition between resistant and susceptible RILs in health and disease independent of environmental factors. These data highlight the role of the genetic constitution in shaping the microbiome, thereby contributing to increased protection or risk of periodontal destruction. Future studies will characterize the underlying causal genetic variants and provide proof for the interrelations with specific bacterial taxa that influence oral health and disease. This may guide important preventive and therapeutic strategies for dental care.

## 4. Materials and Methods 

All experiments were conducted at the Department of Clinical Microbiology and Immunology, Sackler Faculty of Medicine, Tel-Aviv University (TAU), Israel. All experimental mice and protocols were approved by the Institutional Animal Care and Use Committee of TAU (approval number: M-11-026), which adheres to the Israeli guidelines that are in accordance with the recommendations in the Guide for the Care and Use of Laboratory Animals, National Institute of Health (NIH), USA (National Academies Press; 8th edition). Full details of the CC lines were reported previously [31].

### 4.1. Bacterial Cultivation

The strains *P.g* 381 and *F.n. 1594* were grown in peptone yeast extract containing hemin and vitamin K (Wilkins Chalgren broth, Oxoid Ltd., Hampshire, UK) in an anaerobic chamber with 85% N_2_, 5% H_2,_ and 10% CO_2_ followed by three washes in phosphate-buffered saline PBS. The bacterial concentration was measured using spectrophotometry standardized to OD_650_ nm = 0.1 for *P.g*., corresponding to 10^10^ bacteria/mL [32,33], and OD_660_ nm = 0.26 for *F.n*., corresponding to 10^9^ bacteria/mL.

### 4.2. Assessment of Lines Susceptibility to Alveolar Bone Loss

**Figure 7:**
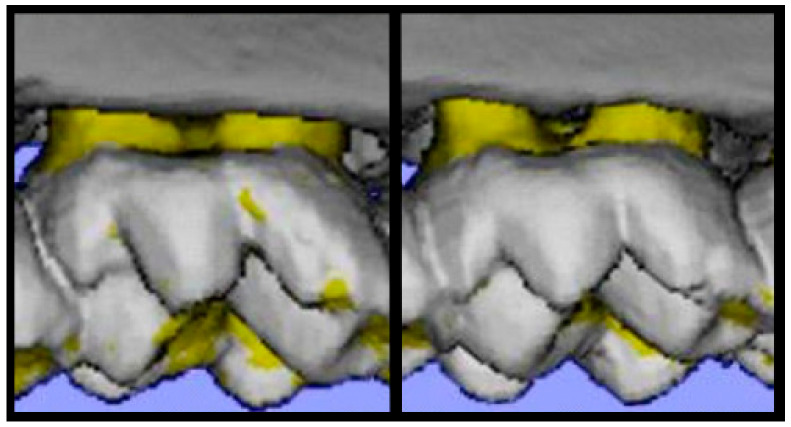
Micro-computed tomography (μCT) Left frame, expanded view of uninfected left hemi-maxilla. Right frame, expanded view of post-infection left hemi-maxilla. White, enamel; yellow, dentin, and cementum; gray, alveolar bone. Horizontal resorption is measured as the distance from the cementoenamel junction (CEJ, the line between the yellow and gray colors) to the alveolar bone crest.

To assess the susceptibility of the CC lines to experimental periodontitis, we tested the alveolar bone changes among seven different lines (IL72, IL211, IL188, IL1912, IL2126, IL2513, and IL3912) after oral mixed infection by quantifying the alveolar bone volume using the micro-CT (μCT) technique. Figure 7 demonstrates the μCT assessment of two hemi-maxilla of two mice with different susceptibility statuses.

In total, we used 87 mice: 43 mice for the control and 44 mice for the challenge (a minimum of 5 mice were used per line in each group). Details are shown in Table 1:

### 4.3. Oral Mixed Infection Model and Micro-Computerized Tomography (CT) Analysis

Mice were treated with sulfamethoxazole (0.8 mg/mL) in drinking water for a continuous period of 10 days, followed by an antibiotic-free period of three days. Mixed cultures of *P.g.* and *F.n.* (400 uL of 10^9^ bacteria/mL for each mouse) were prepared. Next, the infected groups were treated with the bacteria, while the control groups were treated with PBS and 2 carboxymethy cellulose at days 0, 2, and 4. Forty-two days post-infection, mice were euthanized after complete anesthesia, using xylisine (Sedaxylan) and ketamine (Clorketam). The infection challenge of the CC lines was carried out at the small animal facility at Tel-Aviv University (TAU). Susceptibility to alveolar bone loss-induced mixed infection among the selected lines was determined as previously described by our group. Briefly, maxillary jaws were harvested for micro-computed tomography (μCT) analysis, and alveolar bone loss was calculated proportionally to the alveolar bone volume of a control group (non-infected) of the same line [22].

### 4.4. Oral Microbiome Collection 

Of the scanned 87 mice, 53 were used in the microbiome analysis: 53 oral swap samples and 53 from 7 different CC lines were collected and used for the microbiome assessment based on their susceptibility status (i.e., susceptible vs. resistant). Briefly, the biological samples for each status were collected at three points during the experiment and pooled for analysis. The oral samples were collected from mice at three groups/time points (point 1, day 0 (without infection); point 2, 14 days after infection; and point 3, 42 days after infection). Samples were collected using dry fine-tipped swabs and sterile paper points. The oral cavity of each mouse was swabbed for ~50 s (starting on the tongue, followed by buccal mucosa and gingiva). The swab was placed in empty Eppendorf tubes, swab handles were cut with sterile scissors, and samples were stored at −80 °C for further processing.

### 4.5. DNA Extraction and 16s rRNA Sequencing 

The control (time point 1) and infected oral samples (time points 2 and 3) were used for DNA extraction using the Gene AllExgene^TM^ cell SV mini (100p/Catalog No:106-101/LotNo: 10618A26008) Kit. In total, 53 samples (15 samples at point 1, 17 at point 2, and 21 at point 3; details are shown in Table 2) were included and sequenced with the 16S sequencing library preparation protocol [34]. Briefly, after extracting pure DNA from the different samples, published primers were used to amplify the V3-V4 region of the bacterial 16S rRNA gene. PCR products were visualized following electrophoresis in agarose and staining with Gel Red™ to confirm a positive yield for each sample. Library preparation followed the Illumina library preparation protocol, with the following primers: forward CCTACGGGNGGCWGCAG and reverse GACTACHVGGGTATCTAATCC. Sequencing was carried out using Illumina Miseq at the Hadassah Medical School facility.

### 4.6. Microbiome Analysis

Reads with poor quality were filtered out, and after two filtration steps, the reads were reduced from 5194 to 67 taxa. Sequencing adapters and barcodes were trimmed, and each set of paired-end reads was merged into a single sequence (based on overlap). The sequences were assigned to individual samples with barcodes. Metagenomics workflow classified organisms from V3 and V4 amplicon using a database of 16S rRNA data. The classification is based on the Greengenes database (http://greengenes.lbl.gov/; accessed on 20 May 2020).

Data analysis was performed using the statistical software package MICC, v2 to produce operational taxonomic units (OTUs) at the genus level. Next, the phyloseq package of R was used to compute the number of OTUs and the Shannon diversity index, which is used to calculate the total number of species-level phylotypes. A one-way analysis of variance was used to test the significance of the difference between the treated groups. After significance was established, the inter-group differences were tested for significance using the *t*-test with the Shannon index for multiple testing. The level of significance was determined at a corrected significance threshold of *p* < 0.05.

### 4.7. Statistical Analysis

µCT data were analyzed using the statistical software package SPSS version 21. An analysis of variance (ANOVA) was performed to test the differences in response between the different CC lines. The level of significance was determined at *p* < 0.05. All results were presented as mean values and standard error of the mean.

## Figures and Tables

**Table 1:** List of used CC mice in the experiment.

CC-Line	Infection	Control	Total
IL-72	5	5	10
IL-2126	5	5	10
IL-2513	8	5	13
IL-3912	5	6	11
IL-188	7	6	13
IL-211	6	6	12
IL-1912	8	10	18

**Table 2:** The table lists the details of the collected samples for 16s rRNA sequencing.

	Resistant Group	Susceptible Group	Total
Control (Point 1—day 0; before infection)	11 mice	4 mice	15 mice
Point 2—14 days after infection	13 mice	4 mice	17 mice
Point 3—42 days after infection	14	7 mice	21 mice
			53 mice

## Data Availability

The data presented in this study are available on request from the corresponding author.
